# Implementation of a low-flow intervention at a veterinary teaching hospital: an environmental initiative

**DOI:** 10.1038/s41598-026-60933-9

**Published:** 2026-07-20

**Authors:** Dany Elzahaby, Olivier Louis Levionnois, Claudia Spadavecchia

**Affiliations:** Anaesthesiology and Pain Therapy Section, Department of Clinical Veterinary Medicine, Vetsuisse Faculty, University of Bern, Bern, Switzerland

**Keywords:** Low flow, Sustainability, Veterinary, Environment, Climate, Greenhouse gases, Diseases, Environmental sciences, Health care, Medical research

## Abstract

Inhalational anaesthetics such as isoflurane and sevoflurane are widely used in veterinary medicine, but are potent greenhouse gases that contribute substantially to climate change. Low-flow anaesthetic techniques are recommended to minimise their environmental impact, yet their adoption in veterinary practice remains uncertain. This study evaluated the environmental benefits of implementing a standardised low-flow anaesthesia protocol at a veterinary teaching hospital as part of a sustainability initiative. Anaesthetic records from 120 procedures before and 120 after the intervention were analysed to compare FGF rates and volatile agent use during the maintenance phase. The intervention included staff education, email correspondence, visual aids, and a standardised low-flow protocol. Post-intervention, mean FGF decreased by 52% (from 1.27 to 0.61 L/min), corresponding to a proportional 52% reduction in greenhouse gas emissions (from 4.24 to 2.03 kg CO_2_e/h). This equated to a decrease in average car mileage emissions from 31.65 to 15.17 km driven per hour of anaesthesia. Survey responses from anaesthesia staff (*n* = 21) indicated strong support for the protocol. Targeted education and standardised protocols can meaningfully reduce inhalant use and emissions in veterinary anaesthesia. Broader adoption of similar initiatives could yield considerable environmental benefits across the veterinary profession.

**Supplementary Information:**

The online version contains supplementary material available at 10.1038/s41598-026-60933-9.

## Introduction

Inhalational anaesthetic agents such as isoflurane and sevoflurane are widely used in both human and veterinary medicine to facilitate a broad range of medical procedures. After administration, these agents are typically scavenged and released into the atmosphere, where they act as potent greenhouse gases, contributing to climate change^1^. In human medicine, current recommendations encourage prioritising total intravenous anaesthesia (TIVA) over inhalational anaesthesia where clinically appropriate^2–4^. In contrast, veterinary practice continues to rely heavily on volatile agents^5^, which are estimated to contribute up to 21% of total surgical carbon emissions^6^.

Given this reliance, reducing inhalant consumption wherever feasible is an important aspect of environmental stewardship in veterinary practice. Inhalant consumption is primarily determined by vaporiser settings and fresh gas flow (FGF) rates, with FGF exerting the greatest influence^7^. FGF rates can be categorized as low (0.5–1 L/min), minimal (0.25–0.5 L/min), or metabolic (based on oxygen consumption), all of which are achievable when using a circle breathing system^1^. Lowering FGF leads to proportional reductions in vaporiser output and associated anaesthetic gas emissions, while also improving heat and moisture retention and reducing costs^1,8–10^. Consequently, low-flow techniques are recommended and increasingly regarded as best practice^1^. Despite this, only 49% of surveyed anaesthetists in human medicine report routinely using FGF rates below 1 L/min^11^. Whether similar practices are followed in veterinary medicine is unclear.

The primary aim of this study was to compare average FGF rates during the maintenance phase of anaesthesia before and after the implementation of a low-flow protocol at a veterinary teaching hospital, and to quantify the associated environmental impact. A secondary aim was to survey anaesthetists post-intervention to assess their perceptions and acceptance of the low-flow protocol.

## Materials and methods

### Study design and clinical setting

This descriptive, observational study was conducted at a small animal veterinary teaching hospital. The study compared inhalational anaesthetic consumption, and its associated environmental consequences, between two time periods: pre- and post-intervention. At this hospital, anaesthesia is supervised by diplomates of the European College of Veterinary Anaesthesia and Analgesia (ECVAA) or the American College of Veterinary Anesthesia and Analgesia (ACVAA), and is performed by students, rotating interns, technicians, anaesthesia residents, or diplomates. Comprehensive monitoring is employed for all anaesthetic procedures, including continuous measurement of anaesthetic gas concentrations.

## Data collection: pre- and post-intervention

Anaesthetic records from June 2024 served as the pre-intervention dataset. During the pre-intervention period, there was no enforced standard for flow rates, though low-flow techniques were generally recommended. Anaesthetic records from the hospital’s central electronic database were reviewed. Records were included if they met the following criteria:

- Use of isoflurane or sevoflurane
- Use of a rebreathing system
- Minimum of 60 min of inhalational anaesthesia; an additional 15 min required if a machine switch occurred
- Complete documentation of the anaesthetic procedure
- Continuous anaesthetic management by the same primary anaesthetist

Data were collected consecutively until 120 protocols were included in both pre- and post-intervention groups. Anaesthetic details including duration, anaesthetist qualification, as well as vaporiser and FGF settings per 5-minute intervals were transcribed into Microsoft Excel. Patient data including weight and species were also recorded. Anaesthetic records from June 2025 served as the post-intervention dataset and were collected in the same manner after the intervention.

## Intervention

The intervention ran from January 2025 to May 2025. During this time, a standardised low-flow rate protocol was introduced in consultation with anaesthesia specialists at the hospital (Table 1). It specified initial and maintenance FGF rates and vaporiser settings based on patient weight (< 10 or > 10 kg). The protocol was developed taking into consideration factors such as patient size, circuit volume, metabolic oxygen requirements, minor leaks, and flowmeter/vaporiser accuracy.

Initial FGFs were calculated using the equation $\:T=\frac{{V}_{BS}}{{\dot{V}}_{F}}$, where *T* is the time constant (minutes), *V*_*Bs*_ is system volume (litres), and $\:\dot{V}_{F}$ is the FGF (L/min)^12^. For the circle breathing circuits and anaesthesia machines used in this setting, a system volume of approximately 5 L was assumed. At a FGF of 4 L/min, it would take approximately 4 min to reach three time constants, corresponding to 95% equilibration between the vaporiser setting and the inhalant concentration within the breathing system.

Staff were notified of the intervention verbally, with reinforcement provided through email correspondence and oral presentations. Visual aids were also utilized to promote adherence to the protocol and to raise awareness of the environmental impact of inhalational anaesthesia. All relevant staff were encouraged to follow the protocol outlined in Table 1. Where a different inspired oxygen fraction (FiO₂) was required, clinicians were instructed to adjust the oxygen-to-air ratio while maintaining the total flow rate. Deviations from the protocol were permitted when clinically indicated.

**Table 1:** Low-flow anaesthesia protocol - recommended flow rates and vaporiser settings for patients weighing < 10 kg and > 10 kg.

Parameter	Patients < 10 kg	Patients > 10 kg
Initial Flow rate (L/min)*	O₂: 1 L/min Air: 1 L/min	O₂: 2 L/min Air: 2 L/min
Initial Vaporiser Setting*	Isoflurane: 2% Sevoflurane: 3%	Isoflurane: 2% Sevoflurane: 3%
Maintenance Flow Rate	O₂: 0.2 L/min Air: 0.2 L/min	O₂: 0.3 L/min Air: 0.3 L/min

*Initial settings were intended for use at the start of inhalational anaesthesia and expected to transition to maintenance settings within 15 min.

## Volatile anaesthetic consumption

Mean FGF rates were calculated for each anaesthetic event, which comprised the maintenance anaesthetic period. For a representative sample, the initial 15 min of each anaesthetic procedure were excluded. For procedures involving a change in anaesthetic machine, an additional 15-minute exclusion period was applied post-switch. A minimum of 45 min of maintenance inhalational anaesthesia (post-exclusion) was required to calculate a valid mean FGF and mean vaporiser setting for a given procedure. Mean FGF and mean vaporiser settings were then calculated using all eligible intervals for each case. These values, and corresponding anaesthetic durations, were compared between pre- and post-intervention periods. The resulting data were subsequently used to estimate the environmental impact of inhalant agent use.

## Environmental impact calculation

For each anaesthetic event, volatile agent consumption was estimated using the following formula^7^:

$$\text{Fluid Volatile Agent (mL)} = \frac{\text{Mean Fresh Gas Flow (mL/min)} \times \text{Mean Agent Conc. (Vol}\%) \times \text{Anaesthetic Duration (mins)}} {\text{Saturated Gas Volume (mL)} \times 100 ~(\mathrm{Vol} \%)}$$

Volatile agent volumes were converted to mass (g) using agent-specific densities for isoflurane and sevoflurane, 1.49 g/mL or 1.53 g/mL, respectively^7^. Carbon dioxide equivalents (CO_2_e) were calculated using the 100-year global warming potential (GWP_100_) for isoflurane and sevoflurane, 510 or 130, respectively^13^:

CO_2_e (t) = Weight of Gas (t) x GWP_100_

CO₂e values were compared between the two study periods to assess changes in environmental impact. To improve reader relatability, corresponding car mileage equivalents were calculated using European average emissions for petrol passenger vehicles (134 g of CO₂e/km driven)^14^.

### Survey

At the conclusion of the post-intervention period, anaesthetists were anonymously surveyed to evaluate the perceived feasibility of the low-flow protocol and their likelihood of continuing low-flow anaesthesia in future practice. Survey questions are provided in Supplementary 1. All methods were performed in accordance with the relevant guidelines and regulations.

## Results

The pre-intervention period included anaesthetic procedures performed in June 2024, while the post-intervention period included anaesthetic procedures from June 2025. Anaesthetic protocols meeting the inclusion criteria were consecutively collected until 120 cases were obtained for each period. The intervention itself was implemented between January and May 2025, and consisted of email correspondence, the introduction of a low-flow protocol (Table 1), visual aids, and oral presentations. Anaesthetic details including species, patient weight, duration of maintenance anaesthesia and use of isoflurane or sevoflurane between both groups are summarized in Table 2.

**Table 2:** Summary of anaesthetic protocol parameters in the pre- and post-intervention periods.

	Pre-Intervention	Post-intervention
Anaesthetics analysed	120	120
Canine (no. of patients)	85	90
Feline (no. of patients)	35	30
Isoflurane (no. of procedures)	107	95
Sevoflurane (no. of procedures)	13	25
Mean Weight (kg)	15.6	15.9
No. Patients < 10 kg/ > 10 kg	52/68	59/61
Mean Duration (mins)	136.2	111.8

## Volatile anaesthetic consumption & environmental impact

In the pre-intervention period, mean FGF rate was 1.27 L/min, while post-intervention, it was 0.61 L/min, reflecting a 52% overall reduction. While there was an increase in mean vaporiser settings for both isoflurane and sevoflurane, overall environmental emissions were reduced from 4.24 to 2.03 kg CO_2_e/h, reflecting a 52% reduction during the maintenance phase. Prior to the intervention, mean FGF exceeded 1 L/min in 60% of cases, compared to only 8% of cases after the intervention. Values and corresponding car mileage equivalents between periods are summarized in Table 3.

**Table 3:** Comparison of anaesthetic settings and estimated environmental impact of isoflurane and sevoflurane before and after intervention (*n* = 240 anaesthetic events).

	Pre-Intervention	Post-Intervention
Anaesthetic Settings		
Mean Isoflurane Vaporiser Setting (%)	1.51	1.63
Mean Sevoflurane Vaporiser Setting (%)	1.90	2.16
Mean FGF (L/min)	1.27	0.61
Environmental Impact		
Mean kg of CO_2_e/h (Isoflurane)	4.62	2.37
Mean kg of CO_2_e/h (Sevoflurane)	1.09	0.74
Overall Mean kg of CO_2_e/h	4.24	2.03
Mean Car Mileage Driven (km driven per hour of anaesthesia)	31.65	15.17

**Fig. 1:**
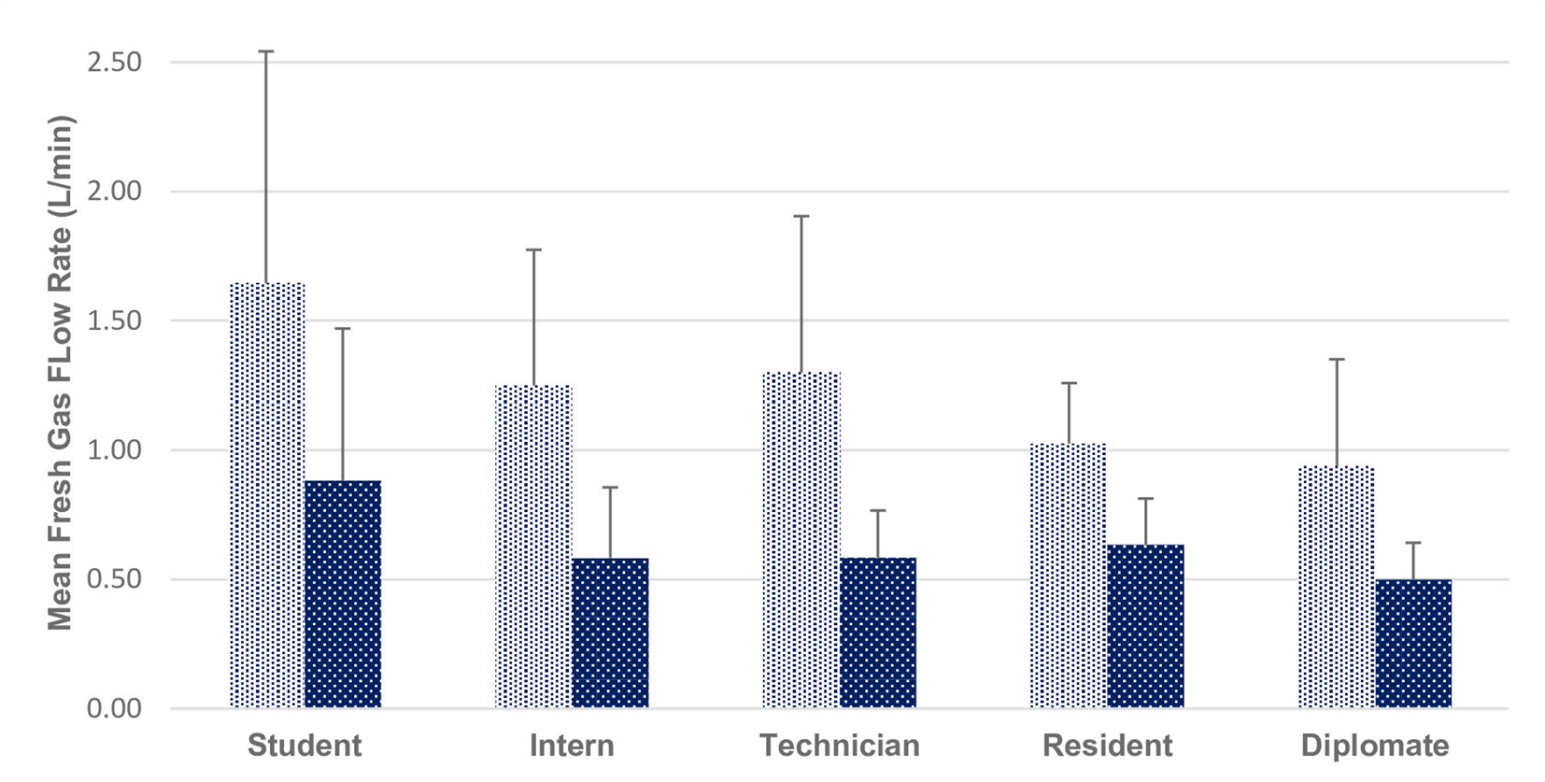
Mean FGF rates grouped according to qualifications. During the pre-intervention period (lightly shaded bars), students performed 10 cases, interns 61, technicians 34, residents 12, and diplomates 3. In the post-intervention period (darkly shaded bars), students performed 8 cases, interns 49, technicians 50, residents 11, and diplomates 2. Vertical bars indicate the standard deviations.

Mean FGF rates were also analysed against anaesthetist qualifications and compared for each period (Fig. 1). Prior to the intervention, mean FGF rates ranged between 0.94 and 1.64 L/min, compared to 0.5 to 0.88 L/min post-intervention.

### Survey

The survey was emailed to 28 anaesthesia staff members who had the opportunity to trial the low-flow anaesthesia protocol, with 21 responses received. Respondents included 8 interns, 6 technicians, 4 residents, and 3 diplomates. All survey respondents found the protocol practical and indicated they were likely to continue using it in future practice. Four of the 21 clinicians reported deviating from the protocol, two preferred lower flow rates, while two preferred slightly higher rates than those recommended. The latter group stated that their preference was primarily due to habit and comfort, and that they would aim to follow the protocol more closely in the future. A summary of the survey responses is presented in Table 4.

**Table 4:** Summary of survey responses following the post-intervention period. Survey respondents included 8 interns, 6 technicians, 4 residents, and 3 diplomates. Of the 4 respondents who did not adhere to the protocol, 2 reported preferring lower FGFs than recommended, and 2 reported preferring higher FGFs.

	Survey respondents, *n* = 21 (%)
Experience in anaesthesia:	
< 1 year	2 (9.5)
1–2 years	6 (28.6)
2–3 years	1 (4.8)
3–6 years	4 (19)
> 6 years	8 (38.1)
Routine FGF prior to the intervention:	
< 0.4 L/min	2 (9.5)
0.4–0.8 L/min	9 (42.9)
> 0.8 L/min	10 (47.6)
Time taken prior to switching to maintenance flow rates:	
< 5 min	3 (14.3)
5–10 min	9 (42.9)
10–15 min	7 (33.3)
> 15 min	2 (9.5)
Protocol compliance and feedback:	
Respondents that adhered to the protocol	17 (81)
Respondents that found the protocol practical to use	21 (100)
Respondents likely to use the protocol in future practice	21 (100)

## Discussion

Implementation of the low-flow intervention in the small animal teaching hospital led to a 52% reduction in FGF and a corresponding 52% decrease in anaesthetic gas emissions. This equates to reducing the carbon footprint of each hour of anaesthesia from 31.65 km to 15.17 km of car travel. Importantly, these outcomes were achieved without new infrastructure or equipment, relying solely on staff education, existing resources, and adjustments to clinical practice. The results reflect broad compliance with the low-flow protocol among anaesthesia staff. However, based on the distribution of patient sizes in the study population, complete adherence to the protocol could theoretically have achieved up to a 61% reduction in FGF rates. Interestingly, this value closely aligns with the 63% reduction predicted in a prior theoretical model^15^. The shortfall may reflect practical constraints, clinician variability, or insufficient exclusion time post-induction. Nonetheless, the reductions attained represent a meaningful advance toward more sustainable anaesthetic practices in this setting.

The low-flow protocol was developed with several key considerations. Initially, high FGFs were recommended following induction to rapidly achieve target end-tidal inhalant concentrations within a reasonable time. Thereafter, the goal was to implement the lowest practical flow rate that ensured both patient safety and clinician compliance. Technical constraints influenced protocol design. The hospital’s TEC™ series vaporisers (GE Healthcare TEC™ 5, 7 and 850) are calibrated for safe and accurate anaesthetic delivery at flows above 200 mL/min^16^, setting this value as the lower safety limit.

Ensuring adequate oxygen delivery to meet metabolic demands during anaesthesia was also a key consideration. In awake animals, whole body oxygen consumption (V̇O₂, mL/min) can be estimated using Brody’s formula: V̇O₂ = 10.5xW^0.75^, where *W* is bodyweight in kg^17^. Based on this, a 10 kg animal requires approximately 5.9 mL O_2_/kg/min (59 mL/min), and a 50 kg animal about 3.5 mL O_2_/kg/min (175 mL/min), illustrating the inverse relationship between metabolism and bodyweight. It is also generally accepted that V̇O₂ is reduced in anaesthetised patients^18^. Experimental studies have reported V̇O_2_ values of 5–6 mL O_2_/kg/min in awake dogs, decreasing to around 3.5 mL O_2_/kg/min under halothane anaesthesia^19^, and approximately 5 mL/kg/min under isoflurane or sevoflurane anaesthesia^20^. While direct measurements in cats are lacking, a conservative estimate of 5 mL O₂/kg/min was applied to both species while designing the protocol. It is important to recognize that V̇O_2_ varies with anaesthetic protocol, species, patient size, individual variation, and underlying disease, rendering any general estimate inherently fallible^21^. Nevertheless, given the mean patient body weight in this study (15.6 and 15.9 kg), it is likely that most patients received oxygen in excess of their true metabolic requirements during anaesthesia.

Additionally, matching FGF to metabolic oxygen requirements in small patients may necessitate very low oxygen flow rates. At these levels, flows may approach or fall below the accurate operating range of standard flowmeters and are increasingly vulnerable to circuit leaks, gas sampling, and vaporiser imprecision at low settings. In practice, this would require delivery of pure oxygen and an FiO_2_ approaching 100%, which is associated with adverse effects, particularly absorption atelectasis^22^, although its clinical relevance may be minimal in short veterinary procedures. Nevertheless, to mitigate this potential risk, an oxygen/air mixture was selected, requiring higher FGFs than those dictated by metabolic demand alone.

During the pre-intervention period, FGF rates varied by anaesthetist qualification, ranging from 0.94 to 1.64 L/min, with students using the highest rates and diplomates the lowest (Fig. 1). These elevated rates likely reflect ingrained habits and comfort levels, as indicated by 2 of the 21 survey respondents who reported using higher FGFs than those recommended in the protocol, citing greater comfort with these settings. Notably, although students operate under the supervision of diplomates within the teaching hospital, they consistently maintained the highest settings. This pattern suggests possible oversight, gaps in training, or that low-flow techniques may be deprioritized amid competing clinical teaching objectives. Interpretation is limited by unequal group sizes, but most clinicians, regardless of qualification, exceeded recommended low-flow rates, with considerable variation across groups. Following implementation of the standardised protocol, FGF rates became markedly more consistent, ranging from 0.58 to 0.63 L/min when excluding students, and up to 0.88 L/min including students. This reduction in variability highlights the effectiveness of standardised protocols in aligning clinical practice, provided that there is adequate staff engagement and compliance.

Although the benefits of low-flow anaesthesia generally outweigh its limitations, several practical considerations should be acknowledged. Foremost is the need for appropriate equipment, specifically, rebreathing systems and adequate anaesthetic monitoring. In the absence of anaesthetic gas monitoring, routine vaporiser calibration and conservative vaporiser settings become essential to minimise the risk of dosing errors or inadvertent overdose. Consequently, implementation of low-flow techniques can prove challenging in resource-limited veterinary practices compared with teaching hospitals or referral centres. Nevertheless, the economic advantages of low-flow anaesthesia, particularly the long-term reduction in anaesthetic agent and carrier gas consumption, may help justify investment in staff training and suitable anaesthetic equipment. Ultimately, recognizing these equipment requirements and limitations allows clinicians to implement low-flow techniques safely within the constraints of their specific practice settings.

The suitability of rebreathing systems also depends on patient size. Veterinary literature shows some variability in recommended thresholds, although most sources consider them appropriate for patients weighing more than 2.5–3 kg^23,24^. In contrast, anaesthetists in this setting typically elect to use rebreathing systems for patients weighing more than 1 kg, highlighting this inconsistency. This difference may reflect advances in anaesthetic equipment, improvement in monitoring capabilities, or increased clinician familiarity and confidence with these systems.

Other drawbacks are relatively minor. For instance, changes in anaesthetic depth occur more slowly at lower FGF rates, occasionally necessitating a temporary increase in FGF to accelerate anaesthetic delivery. Clinicians accustomed to rapid adjustments in end-tidal anaesthetic concentrations may need to adapt their expectations or briefly alter flow or vaporiser settings to achieve the desired effect. System leaks also become more apparent at lower flows; however, this heightened sensitivity can be beneficial by enabling earlier leak detection and reducing occupational exposure to inhalational anaesthetics. Finally, there is a risk of delivering an inadequate oxygen mixture^25^, which can be effectively mitigated with appropriate monitoring equipment and vigilant clinical oversight.

Sevoflurane was also used with low FGF rates within this study, with 100% of survey respondents reporting that they did not adjust FGF based on whether isoflurane or sevoflurane was used. This aligns with a multisite analysis of sevoflurane use in dogs, where clinicians routinely administered it at FGF rates as low as 0.5 L/min^26^. Since its introduction, sevoflurane has been shown to interact with alkaline CO_2_ absorbers, resulting in the formation of an olefin molecule known as Compound A. Experimental studies have demonstrated that prolonged exposure to high concentrations of Compound A can cause nephrotoxicity in rats^27,28^. However, these effects have not been replicated in clinical studies involving humans or in dogs^29–32^. Despite this, the U.S. Food and Drug Administration (FDA) recommends maintaining FGF rates above 2 L/min to minimize exposure to Compound A^33^. Conversely, the American Society of Anaesthesiologists (ASA) states, “*The ASA has evaluated current scientific studies and concludes there is no reasonable evidence to support a lower limit of fresh gas flow when using sevoflurane*”^33^. Given the widespread use of sevoflurane at low FGFs, there appears to be little concern regarding Compound A in the clinical setting. Accordingly, the low-flow protocol implemented in this study was not modified based on the choice of inhalant agent used. Owing to its environmental benefits, greater emphasis should be placed on promoting sevoflurane use in most settings^1,5,13^.

According to the survey, nearly half of the survey respondents (10 out of 21) reported using FGF rates above 0.8 L/min prior to protocol implementation, aligning with the finding that 60% of pre-intervention cases exceeded the low-flow threshold (1 L/min). Post-intervention, all survey respondents considered the low-flow protocol practical and indicated they were likely to continue using it in future practice. This was reflected in the observed reduction in mean FGF, and the fact that only 8% of post-intervention cases exceeded the low-flow threshold. These outcomes support one of the primary goals of the protocol, to promote clinician compliance through simplicity and ease of use. Given that higher FGFs were likely driven by habit rather than clinical necessity, it will be interesting to observe whether this trend persists over time, particularly in the absence of ongoing reinforcement. Beyond reducing FGF, the initiative may also foster greater awareness of habitual practices, potentially encouraging more sustainable approaches in other areas of veterinary care^34^.

This study has several limitations, most of which relate to potential human error. Protocols were completed by clinicians working in high-pressure, time-sensitive environments, where accurate record-keeping and time logging may be challenging. Environmental emission estimates were calculated using a formula that depends heavily on precise records^7^; an alternative approach could have been to weigh vaporisers before and after anaesthesia, or to weigh the isoflurane bottle before and after filling, as previously described^5^. The study also does not account for the initial flow rate following induction or after any machine changes. Nonetheless, the aim of this study was to compare environmental impact between two periods following an environmental initiative, using a consistent methodology, rather than quantifying absolute CO₂ emissions. Finally, the effects of PIVA, locoregional techniques, or patient ASA status were not accounted for and may have influenced vaporiser settings, although observed variations were small and generally consistent with the expected effect of reduced FGF rates.

Another limitation lies in the influence of active encouragement and oversight on the success of the intervention. Whether these outcomes will be sustained in the absence of ongoing reinforcement remains uncertain. Furthermore, the protocol’s simplicity and generalisation could be seen as a drawback. A small proportion of respondents (2 out of 21) reported routinely using lower FGF rates than those recommended in the protocol, suggesting that greater reductions in environmental emissions may be attainable. Nevertheless, despite its simplicity and inherent limitations, the intervention proved both practical and effective in reducing FGF rates. Future iterations might include additional weight brackets, or lower total FGF rates in smaller patients. However, any increase in complexity must be balanced against the potential for reduced clinician compliance.

While the present intervention focused on reducing FGF, this represents only one component of a broader strategy to minimise the environmental impact of anaesthetic practice. Additional strategies include selecting lower-impact inhalant agents, minimising anaesthetic duration where feasible, and considering TIVA or emerging gas capture and recycling technologies, each with its own practical and environmental implications.

## Conclusion

This study demonstrates that the implementation of a standardised low-flow anaesthesia protocol in a veterinary teaching hospital can effectively reduce FGF rates, and consequently anaesthetic gas emissions. The protocol benefited all anaesthetists, with particular value for less experienced staff who may be less familiar or confident with low-flow techniques. While opportunities for further refinement remain, the findings support the feasibility of adopting low-flow techniques in routine veterinary anaesthesia without disrupting clinical workflow or increasing staff burden. These results highlight the potential for significant environmental benefits if similar strategies are implemented in the wider veterinary field. Ultimately, this study illustrates how modest changes in anaesthetic management can yield meaningful environmental benefits, reinforcing the important role of veterinary professionals in promoting sustainable clinical practices.

## Supplementary Information

Below is the link to the electronic supplementary material.

Supplementary Material 1

## Data Availability

The datasets generated and/or analysed during the current study are available in the Zenodo repository: https://doi.org/10.5281/zenodo.16941218, or available from the corresponding author upon reasonable request.
