# Supplementary material for: Implementation of a low-flow intervention at a veterinary teaching hospital: an environmental initiative
Source: Sci Rep. 2026 Jul 20;16:25493. doi: 10.1038/s41598-026-60933-9 (PMC13476265; doi:10.1038/s41598-026-60933-9)
Supplement: Supplementary file 1 [file 41598_2026_60933_MOESM1_ESM.pdf]

Low Flow Anaesthesia Survey

Thank you for taking the time to complete this survey, which supports our study on low-flow anaesthesia. It should take approximately 3 to 5 minutes. Responses are completely anonymous, so we encourage you to answer honestly and critically.

The questions are intended to gather feedback on the low-flow protocol that was introduced earlier this year. Reminder:

- For patients <10 kg: O_2 0.2 L/min + Air 0.2 L/min: Total Flow 0.4 L/min
- For patients >10 kg: O_2 0.3 L/min + Air 0.3 L/min: Total Flow: 0.6 L/min

Please note that we are interested in your feedback on the maintenance phase of anaesthesia, excluding the initial period after connecting to the machine.

Part 1: Before the Low-Flow Protocol

1. What was the most common total fresh gas flow rate you used during the maintenance phase of anaesthesia before the low-flow protocol was introduced? *(Drop-down list)*

- < 0.4 L/min
- 0.4 – 0.8 L/min
- > 0.8 L/min

2. What was the most common oxygen concentration (FiO_2%) you typically used? *(Drop-down list)*

- < 50%
- 50–70%
- > 70%

Part 2: After the Low-Flow Protocol

1. In most cases, did you follow the new flow rate protocol during the maintenance phase of anaesthesia (i.e. 0.4 L/min (0.2 + 0.2 L/min) for patients < 10 kg and 0.6 L/min (0.3 +0.3 L/min) for patients >10 kg)? *(Drop-down list)*

- Yes
- No
    - If you answered “no”:
        - Please state which total fresh gas flow rate you routinely used instead? [Free text box]
        - Please briefly explain why you did not follow the protocol. [Free text box]

2. Did you use a different oxygen concentration (FiO2%) than the one recommended in the protocol *(Drop-down list)*

- Yes
    - If yes, which of the following did you use instead:
        - < 50%
        - 50–70%
        - > 70%
- No

3. How long after induction did you typically reduce the flow rate? *(Drop-down list)*

- Less than 5 minutes
- 5–10 minutes
- 10–15 minutes
- More than 15 minutes

4. Did you adjust the flow rate differently depending on whether isoflurane or sevoflurane was used? *(Drop-down list)*

- Yes
    - If yes, how?
    [Free text box]
- No

5. Did you find the low flow rate protocol practical to use? *(Drop-down list)*

- Yes
- No
  If no, please explain:
  [Free text box]

6. Do you have any suggestions for improving the low flow rate protocol?
   [Free text box]

7. Are you likely to continue to use the low-flow protocol recommended in future practice (i.e. 0.4 L/min (0.2 + 0.2 L/min) for patients < 10 kg and 0.6 L/min (0.3 +0.3 L/min) for patients >10 kg)?

- Yes
- No

Part 3: Background information

8. What is your professional qualification? *(Drop-down list)*

- Student
- Intern
- Technician
- Resident
- Diplomate

- Other
    - If other, please specify

    [Free text box]

9. How long have you been performing anaesthesia? *(Drop-down list)*

- Less than 1 year
- 1–2 years
- 2–3 years
- More than 3 years

10. If you have any other comments at all, please feel free to leave them here, thanks again.

[Free text box]
